# Is dorsal anterior cingulate cortex activation in response to social exclusion due to expectancy violation? An fMRI study

**DOI:** 10.3389/fnevo.2012.00011

**Published:** 2012-07-27

**Authors:** Taishi Kawamoto, Keiichi Onoda, Ken'ichiro Nakashima, Hiroshi Nittono, Shuhei Yamaguchi, Mitsuhiro Ura

**Affiliations:** ^1^Department of Behavioral Sciences, Graduate School of Integrated Arts and Sciences, Hiroshima UniversityHigashi-Hiroshima, Japan; ^2^Faculty of Medicine, Department of Neurology, Shimane UniversityIzumo, Japan; ^3^Preschool Education Section, Nagasaki Women's Junior CollegeNagasaki, Japan

**Keywords:** social exclusion, expectancy violation, anterior cingulate cortex, ventrolateral prefrontal cortex, event-related design, fMRI

## Abstract

People are typically quite sensitive about being accepted or excluded by others. Previous studies have suggested that the dorsal anterior cingulate cortex (dACC) is a key brain region involved in the detection of social exclusion. However, this region has also been shown to be sensitive to non-social expectancy violations. We often expect other people to follow an unwritten rule in which they include us as they would expect to be included, such that social exclusion likely involves some degree of expectancy violation. The present event-related functional magnetic resonance imaging (fMRI) study sought to separate the effects of expectancy violation from those of social exclusion, such that we employed an “overinclusion” condition in which a player was unexpectedly overincluded in the game by the other players. With this modification, we found that the dACC and right ventrolateral prefrontal cortex (rVLPFC) were activated by exclusion, relative to overinclusion. In addition, we identified a negative correlation between exclusion-evoked brain activity and self-rated social pain in the rVLPFC, but not in the dACC. These findings suggest that the rVLPFC is critical for regulating social pain, whereas the dACC plays an important role in the detection of exclusion. The neurobiological basis of social exclusion is different from that of mere expectancy violation.

## Introduction

Human beings are sensitive to the negative aspects of interpersonal relationships, including such experiences as being excluded or ostracized (e.g., Williams et al., 2000; Zadro et al., 2004; Gonsalkorale and Williams, 2007; Williams, 2009). This sensitivity can be interpreted as evolutionarily adaptive (Baumeister and Leary, 1995; Leary and Baumeister, 2000; Williams, 2009). For example, baboon offspring of females who have strong relationships with others have a high probability of survival (Silk et al., 2003). In addition, monkeys subjected to an amygdalectomy show reduced social interaction, are excluded from their groups, and ultimately die (Kling et al., 1970). These findings suggest that mammals that have strong relationships with others in their social groups are more likely to survive than those who do not have such relationships. In order to effectively adapt to social environments that can change quite frequently, human beings have developed monitoring or detection systems that are highly sensitive to social exclusion (Leary and Baumeister, 2000; Pickett and Gardner, 2005).

People can detect quite subtle social exclusion cues, which often evoke aversive feelings. A simple interactive computer-based ball-tossing game called Cyberball (Williams et al., 2000) has been used to manipulate social exclusion in various social psychology and neuroscience investigations (e.g., Eisenberger et al., 2003; Zadro et al., 2004; van Beest and Williams, 2006; Onoda et al., 2009, 2010; Yanagisawa et al., 2011a,b). In this paradigm, two or three ostensible players throw the ball to the participant and to one another, such that the participant can be included in the game or excluded. Previous studies using this paradigm have revealed that social exclusion evokes a negative mood state and participant-perceived detrimental shifts in four fundamental needs: self-esteem, meaningful existence, belonging, and control (e.g., Williams et al., 2000; Zadro et al., 2004; Gonsalkorale and Williams, 2007). These effects occur even when participants realize that the other players are not important figures for them (Zadro et al., 2004; Gonsalkorale and Williams, 2007; Onoda et al., 2009). These findings suggest that people are highly sensitive to being accepted or excluded by others, such that they can detect even the slightest cues of exclusion.

One candidate brain region for the detection and processing of social exclusion is the dorsal anterior cingulate cortex (dACC). Several neuroimaging and computational modeling studies has revealed that the dACC serves as a conflict or discrepancy detector during information processing (e.g., Bush et al., 2000; Botvinick et al., 2001, 2004). Eisenberger et al. (2003) found that social exclusion activated the dACC as compared to social inclusion, even when participants were told that they are being excluded accidently. In addition, dACC activity in response to social exclusion was positively correlated with self-rated social pain. Other studies have replicated the finding of dACC involvement in social exclusion (e.g., Eisenberger et al., 2007, 2011; Krill and Platek, 2009; Onoda et al., 2009, 2010; Dewall et al., 2012). This region is also known to be activated during the experience of physical pain (e.g., Rainville et al., 1997; Sawamoto et al., 2000) and is thought to work as a neural alarm system (Eisenberger and Lieberman, 2004). In contrast, right ventrolateral prefrontal cortex (rVLPFC) activation in response to social exclusion has been shown to correlate negatively with social pain (e.g., Eisenberger et al., 2003; Yanagisawa et al., 2011a,b). This region is known to be involved in the regulation of distress associated with physical pain as well as other negative emotional experiences (e.g., Hariri et al., 2000; Petrovic et al., 2002; Lieberman et al., 2004, 2007). These findings suggest that the rVLPFC plays an important role in the regulation of social pain.

The exact nature of dACC involvement in psychological responses to social exclusion remains unclear. As human beings appear to have a fundamental need to belong (Baumeister and Leary, 1995), many of us expect other people to follow an “unwritten rule” in which they err on the side of including us in social interactions (Bolling et al., 2011b). The experience of social exclusion therefore involves not only an emotional response to the experience (e.g., social pain): there is also an expectancy violation component (Somerville et al., 2006; Bolling et al., 2011b). There is some evidence that dACC activation during social exclusion may reflect cognitive processes as opposed to the direct emotional experience of social pain. Bush et al. (2000) suggested that there is considerable functional differentiation of the ACC. They argued that the dorsal ACC is sensitive to cognitive information, such as that used during conflict monitoring (Botvinick et al., 2004), whereas the ventral ACC (vACC) is sensitive to emotional information. In line with this notion, Somerville et al. (2006) performed an fMRI study using a rejection paradigm, in order to separate the effects of social rejection and expectancy violation. In this paradigm, participants make social judgments and receive positive or negative feedback from others, such that the feedback is either consistent or inconsistent with their expectations. They found that the dACC was sensitive to expectancy violations, whereas the vACC was sensitive to emotional feedback. On the other hand, Bolling et al. (2011b) performed another fMRI study that sought to eliminate the effects of expectancy violation on participants' responses to social exclusion. This study involved participation in two paradigms: Cyberball and Cybershape. In Cybershape, expectancy is violated without the experience of social exclusion. In this paradigm, there was a rule about throws, but one of the computalized players violated the rule continuously. These researchers found greater dACC and vACC activation during exclusion in Cyberball, as compared to rule violation in Cybershape. Hence, the question of whether dACC activation underlies social exclusion or expectancy violation remains unsettled.

The aim of the present experiment was to separate the neurobiological substrate of expectancy violation from that of social exclusion, and to identify the brain regions involved in social exclusion. To achieve these goals, we conducted a Cyberball task that included an additional “overinclusion” condition (Williams et al., 2000; van Beest et al., 2011), in which participants receive a surprisingly large number of ball tosses. In this condition, participants receive the ball at the same frequency as they do not receive the ball in the exclusion condition. An exclusion condition involves an expectancy violation in which participants receive the ball less often than they expect, whereas an overinclusion condition involves an expectancy violation in which they receive the ball more than they expect. In accordance with this, comparing patterns of activation during both conditions allows one to eliminate the effects of expectancy violation by holding expectancies constant across the two conditions. Secondly, we used continuous short blocks of fair play, exclusion, and overinclusion trials. In most prior studies, an exclusion condition block was conducted after the fair play condition was completed. We conducted a continuous block design to prevent the participants from predicting which sequence of trials is coming next. In addition, a previous study found that dACC and VLPFC activations in response to exclusion were more prominent at the beginning of the exclusion experiences than closer to the end of these experiences (Moor et al., 2012). A relatively short period of exclusion is therefore likely to be more suitable for investigating dACC functioning as compared to a longer period. Note that a continuous block design does appear to elicit feelings of exclusion (Bolling et al., 2011b). Finally, we used an event-related design as was recently done in previous studies (Crowley et al., 2009, 2010; Moor et al., 2012). An advantage of this design is that it allows one to remove the effects of “noise” in the form of participant responses that do not involve them feeling excluded while also enabling the researcher to subdivide the conditions into exclusion-related and overinclusion-related events.

If dACC activity in response to social exclusion merely reflects expectancy violation, activity levels in this area should not differ across exclusion-related and overinclusion-related events. However, if activity in this area reflects the processing of exclusion, exclusion-related events should induce higher levels of dACC activity as compared to overinclusion-related events.

## Methods

### Participants

Twenty-two healthy undergraduate students (3 males, 19 females; mean age = 20.7 years, range = 18–24, SD = 1.7; all right-handed) participated in the experiment. They were paid ¥ 2000 for their participation. All participants gave their written informed consent after receiving a detailed deception of the study, which was approved by the Ethnic and Safety Committees of Shimane University.

### fMRI task

Participants were told that they would play a visual-ball tossing game (Cyberball; Williams et al., 2000) via the Internet with two other players while in the scanner. In a manner similar to previous studies (Eisenberger et al., 2003), participants were told that the study was examining the effects of mental visualization, and that they would be playing an Internet ball-toss game on the computer in order to practice these skills. To enhance the credibility of the task and rationale provided, participants were given fictional personal information about the other players (e.g., age, sex). Participants then observed the two other player online via low-definition images on a web page, so that they could become “acquainted” with them before playing the ball-tossing game. In reality, participants played a preset computer program and the false player information was prepared in advance. After instructions were provided, participants played some practice Cyberball (fair play), and completed questionnaires about social pain (Williams et al., 2000; Onoda et al., 2009, 2010) as to assess baseline feelings.

Participants then played Cyberball during an fMRI scan. The two other players were depicted as animated cartoon icons in the upper corners of the screen. The other players automatically threw the ball to each other or to the participant, waiting 1.0–2.0 s (determined randomly) between throws in order to increase the feeling that the participant was indeed playing the game with other individuals. Participants used their left and right index fingers on a response pad to throw the ball to the left or right player.

Participants played Cyberball in 12 continuous blocks of fair play, exclusion, and overinclusion trials (e.g., fair play, exclusion, overinclusion, exclusion, fair play, overinclusion, etc.). Each block consisted of about 25 throws (duration of ~ 45 s per block). During fair play, participants received the ball on one-half of the throws (50%). During exclusion, participants received the ball on one-fifth of the throws (20%), and during overinclusion, participants received the ball on four-fifth of the throws (80%).

On completion of the virtual game, participants completed questionnaires that assessed social pain levels (Williams et al., 2000; Onoda et al., 2009, 2010). These assessed participants' subjective experiences of self-esteem (“I felt liked”), belongingness (“I felt rejected”), meaningfulness (“I felt invisible”), and control (“I felt powerful”) on nine-point scales. To check the game experience manipulation and to measure subjective deviation from the expectancy regarding how often participants should receive the ball (i.e., 50% of the time), we asked participants to recall the percentage of ball throws that went to them (“What percentage of the throws were thrown to you?”; 0–100 %). In addition, we also asked participants to rate feelings of surprise (“I felt surprised during the task”) on a nine-point scale. Both perceived percentage of throws and level of surprise were used as expectancy violation indices. Questionnaires were completed separately for both exclusion and overinclusion conditions.

### fMRI data acquisition

Imaging data were acquired using a Siemens AG 1.5 T scanner. A time course series of 193 volumes per participant was acquired with echo planar imaging sequences (TR = 3000 ms, TE = 50 ms, FOV = 256 mm, matrix size = 128 × 128, 29 slices, thickness = 4 mm, flip angle = 90°). After functional scanning, structural scans were acquired using T1-weighted gradient echo pulse sequences (TR = 12 ms, TE = 4.5 ms, FOV = 256 mm, flip angle = 20°).

### fMRI data analysis

Imaging data were analyzed using SPM8 software (Wellcome Department of Cognitive Neurology, London, UK). The first three volumes of each fMRI run were discarded due to an unsteady MRI signal. Slice timing correction was performed for each set of functional volumes. Each set was realigned to the first volume, spatially normalized to a standard template based on the Montreal Neurological Institute (MNI) reference brain, and finally smoothed using an 8 mm FWHM Gaussian kernel.

**Figure 1:**
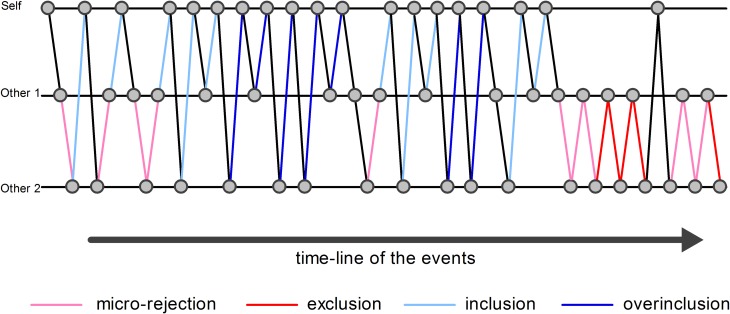
**Examples of analyzed events.** The circles indicate the ball. The pink lines indicate examples of micro-rejection events. The red lines indicate examples of exclusion events. The sky blue lines indicate examples of inclusion events. The blue lines indicate examples of overinclusion events.

An event-related design was modeled, which included “exclusion” event, “micro-rejection” event, “overinclusion” event, “inclusion” event, and response movement (i.e., the button press required to throw the ball to the other player) as regressors (Figure 1). Exclusion event was operationally defined as the events on which participants did not receive the ball more than three consecutive times. Micro-rejection event was operationally defined as the events on which participants did not receive the ball, except for exclusion events (as defined above) and immediately after overinclusion events. Overinclusion event was operationally defined as the occasions on which participants received the ball more than three consecutive times. Finally, inclusion event was operationally defined as the events on which participants received the ball, except for overinclusion events and immediately after exclusion events. Regressor durations were set at 0 s on stimulus onset (i.e., the moment of ball movement).

Random effects analyses of group were conducted using the contrast images generated for each participant. Comparisons of “exclusion vs. micro-rejection” and “overinclusion vs. inclusion” were performed via whole-brain paired *t*-tests. Comparisons of “exclusion vs. micro-rejection” capture processing of both exclusion and expectancy violation, whereas those of “overinclusion vs. inclusion” capture processing of both overinclusion and expectancy violation. The statistical threshold for these *t*-tests was set at an uncorrected *p* < 0.001 and a voxel size of > 10 to maintain a desirable balance between Type I and II errors (Lieberman and Cunningham, 2009). To control for expectancy deviation and direction of the ball, “exclusion—micro-rejection (i.e., exclusion-related event) vs. overinclusion—inclusion (i.e., overinclusion-related event)” comparisons were performed via whole-brain paired *t*-tests. This analysis allowed us to localize regions showing different levels of activation during social exclusion and overinclusion, after excluding the effects of expectancy violation. The statistical threshold for these *t*-tests was set at an uncorrected *p* < 0.001 and a voxel size of >10. Regression analyses were used to detect possible relationships between changes in social pain (i.e., exclusion—overinclusion) and brain activation (i.e., exclusion-related events vs. overinclusion-related events). The threshold for these analyses was set at an uncorrected *p* < 0.001 and a voxel size of >10. All coordinates are reported in MNI coordinate space. The same analysis was also conducted for changes in expectancy violation and brain activation.

## Results

### Subjective ratings

**Figure 2:**
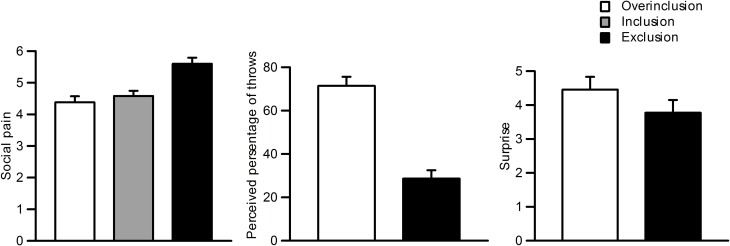
**Subjective ratings during the task.** Left: social pain during inclusion, exclusion, and overinclusion. Middle: surprise during overinclusion and exclusion. Right: perceived percentage of throws during overinclusion and exclusion.

Figure 2 shows self-reported social pain, perceived percentage of throws, and surprise ratings for each session. Repeated measures One-Way ANOVAs were used for statistical analysis of the behavioral data, and Greenhouse–Geisser adjustments were applied. Participants felt more social pain during social exclusion (*M* = 5.6, SD = 0.90) than during fair play (*M* = 4.6, SD = 0.77) and overinclusion (*M* = 4.4, SD = 0.90), *F*_(2, 42)_ = 16.5, ε = 0.93, η^2^_*p*_ = 0.48, *p* < 0.001. There was no significant difference between fair play and overinclusion sessions (*F* < 1).

Participants also reported that more throws went to them during overinclusion (*M* = 71.4%, SD = 19.7) than during exclusion (*M* = 28.6%, SD = 18.2), *t*_(22)_ = 7.07, *p* < 0.001. Furthermore, both conditions significantly differed from the midpoint (i.e., 50%), *t*_(22)_ = −5.21, *p* < 0.001, and *t*_(22)_ = 5.63, *p* < 0.001, respectively. The absolute difference values between the scores for both conditions and the midpoint did not differ (*t* < 1). Participants felt more surprise during the overinclusion session (*M* = 4.4, SD = 1.8) than during the exclusion session (*M* = 3.8, SD = 1.8), *t*_(22)_ = 2.01, *p* = 0.06.

### fMRI analysis

Table 1 shows brain activation comparisons between exclusion and micro-rejection scenarios. Exclusion produced activation in the dACC, insula, and thalamus relative to micro-rejection. Activations of the dorsolateral prefrontal cortex (DLPFC) and caudate nucleus areas were greater for micro-rejection than exclusion. Table 2 summarizes comparisons between overinclusion and inclusion. Overinclusion gave rise to activation in the left visual cortex relative to inclusion. In contrast, the inferior parietal lobule (IPL), superior parietal lobule (SPL), posterior cingulated cortex (PCC), precuneus, right visual cortex, dorsal medial prefrontal cortex (DMPFC), corpus callosum, and premortor cortex showed decreased activation during overinclusion as compared with inclusion.

**Table 1:** **Comparison of brain activations between exclusion and micro-rejection**.

**Brain region**	***x***	***y***	***z***	**size**	***t***
**EXCLUSION > MICRO-REJECTION**					
dACC(24)	0	32	32	36	4.32
R. insula(13)	50	4	−12	17	4.52
	42	12	−8	14	4.12
L. insula(13)	−30	18	−12	33	4.48
L. thalamus	−22	−14	12	27	5.15
**EXCLUSION < MICRO-REJECTION**					
L. DLPFC(4/46)	−48	28	40	25	3.96
R. caudate nucleus	26	−10	26	23	3.79

Notes: L, left; R, right; DLPFC, dorsolateral prefrontal cortex; size: activation voxels, t: t-value. Brodmann's area is provided in parentheses.

**Table 2:** **Comparison of brain activations between overinclusion and inclusion**.

**Brain region**	***x***	***y***	***z***	**size**	***t***
**OVERINCLUSION > INCLUSION**										
L. visual cortex (19)	−34	−58	−4	19	5.34
**OVERINCLUSION < INCLUSION**					
L. IPL (40)	−66	−24	30	65	6.49
R. SPL (7)	24	−40	52	79	5.88
R. IPL (7/40)	20	−54	34	19	4.61
PCC (23/31)	−6	−16	42	64	4.99
	4	6	42	30	4.57
Precuneus (7/31)	−2	−62	46	120	4.64
R. visual cortex (19)	56	−64	−4	63	5.46
R. DMPFC (8)	32	20	42	25	4.9
Corpus callosum	−12	34	18	12	4.24
R. premoter cortex (4)	24	−32	72	29	4.14

Notes: L, left; R, right; IPL, inferior parietal lobule; SPL, superior parietal lobule; PCC, posterior cingulate cortex; DMPFC, dorsomedial prefrontal cortex; size: activation voxels, t: t-value. Brodmann's area is provided in parentheses.

**Figure 3:**
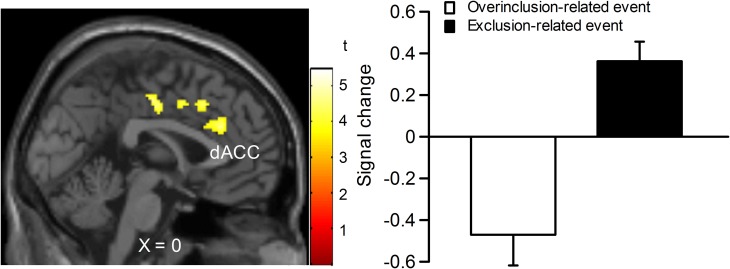
**Comparison of brain activation for exclusion minus micro-rejection in contrast to overinclusion minus inclusion.** Left: sagittal section (X = 0). Right: estimated dACC activity (BA 24: −2, 30, 26; 167 voxels). The threshold for whole brain *t*-test was set at an uncorrected *p* < 0.001, and at voxels > 10.

In order to examine which regions are more activated by social exclusion as compared to social inclusion, after controlling for expectancy violation, we conducted a paired *t*-test comparison of exclusion—micro-rejection and overinclusion—inclusion (Table 3). The contrast of exclusion—micro-rejection vs. overinclusion—inclusion produced significant activation in the dACC (Figure 3) and right ventrolateral prefrontal cortex (rVLPFC), as previously reported in Cyberball studies (Eisenberger et al., 2003). In addition, activation of the ventral and dorsal MPFC, PCC, somatosensory area, premotor cortex, SPL, IPL, and thalamus were also greater for exclusion-related events as compared to inclusion-related events. In contrast, the contrast of overinclusion—inclusion vs. exclusion—micro-rejection gave rise to activation in the bilateral visual cortex.

**Table 3:** **Paired samples *t*-test comparing the exclusion—micro-rejection and overinclusion—inclusion**.

**Brain region**	***x***	***y***	***z***	**size**	***t***
**EXCLUSION—MICRO-REJECTION > OVERINCLUSION—INCLUSION**					
dACC (24/32)	−2	30	26	167	4.99
	0	20	42	44	4.31
R. VLPFC(44)	62	4	4	10	3.82
R. VLPFC(44)/VMPFC(8)	22	44	−12	16	4.69
R. DMPFC (8)	32	20	42	22	5.44
PCC (23/31)	0	−16	44	105	4.62
	2	4	42	41	4.72
R. SSA (4/6)	58	−26	52	10	4.7
R. premorter cortex (4)	30	−26	74	22	4.64
R. SPL (7)	24	−40	52	20	4.6
L. IPL (40)	−66	−22	28	22	4.46
L. thalamus	−26	−26	10	13	4.15
**EXCLUSION—MICRO-REJECTION < OVERINCLUSION—INCLUSION**					
L. visual cortex (19)	−30	−56	−4	10	4.27

Notes: L, left; R, right; VLPFC, ventrolateral prefrontal cortex; VMPFC, ventromedial prefrontal cortex; DMPFC, dorsomedial prefrontal cortex; PCC, posterior cingulate cortex; SSA, somatosensory area; SPL, superior parietal lobule; IPL, inferior parietal lobule; size: activation voxels, t: t-value. Brodmann's area is provided in parentheses.

### Regression analysis

**Figure 4:**
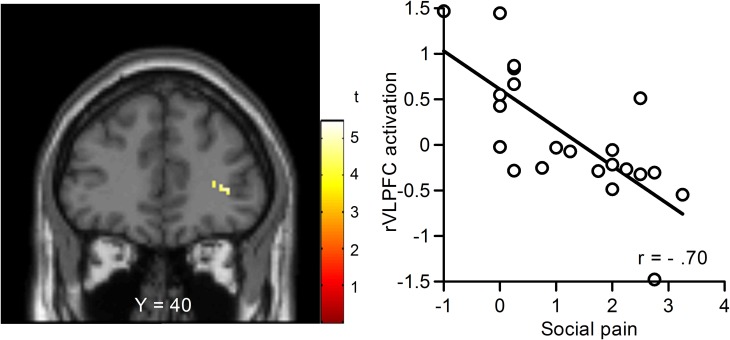
**Relationship between changes in blood-oxygen-level dependent (BOLD) signal and subjective social pain during exclusion.** Left: Coronal section (Y = 40). Right: Scatter plots of rVLPFC activity and social pain. The threshold for these analyses was set at an uncorrected *p* < 0.001 and voxels > 10.

We performed regression analyses to determine the brain regions involved in the perception and modulation of social pain (Table 4). Increases in self-reported pain upon exclusion (the value of social exclusion—overinclusion) were positively correlated only with increases in the corpus callosum. On the contrary, rVLPFC activation was negatively correlated with increases in self-reported pain upon exclusion (*r* = −0.70, *p* < 0.001; Figure 4). In addition, activations of the corpus callosum, IPL, and temporal poles were negatively correlated with increases in self-reported pain upon exclusion. We also conducted regression analyses to determine the brain regions involved in expectancy violation (Tables 5, 6). There were no statistically significant correlations between dACC activation and expectancy violation indices.

**Table 4:** **Regression analyses between brain activation and social pain**.

**Brain region**	***x***	***y***	***z***	**size**	***t***
**POSITIVE CORRELATION BETWEEN CHANGES OF BRAIN ACTIVATION IN ΔEXCLUSION—MICRO-REJECTION > OVERINCLUSION—INCLUSION AND SOCIAL PAIN IN ΔEXCLUSION—OVERINCLUSION**					
Corpus callosum	−12	26	18	15	4.51
**NEGATIVE CORRELATION BETWEEN CHANGES OF BRAIN ACTIVATION IN ΔEXCLUSION—MICRO-REJECTION > OVERINCLUSION—INCLUSION AND SOCIAL PAIN IN ΔEXCLUSION—OVERINCLUSION**					
R. VLPFC (44)	38	40	4	19	4.16
Corpus callosum	14	−12	38	10	4.47
R. IPL (7/40)	50	−38	38	14	4.47
R. TP (38)/STS (21/22)	56	−6	−26	26	4.64

Notes: R, right; VLPFC, ventrolateral prefrontal cortex; IPL, inferior parietal lobule; TP, temporal pole; STS, superior temporal sulcus; size: activation voxels, t: t-value. Brodmann's area is provided in parentheses.

**Table 5:** **Regression analyses between brain activation and perceived percentage of the throws**.

**Brain region**	***x***	***y***	***z***	**size**	***t***
**POSITIVE CORRELATION BETWEEN CHANGES OF BRAIN ACTIVATION IN ΔEXCLUSION—MICRO-REJECTION > OVERINCLUSION—INCLUSION AND PERCEIVED PERCENTAGE OF THE THROWS**					
L. thalamus	−26	−20	14	12	4.55
**NEGATIVE CORRELATION BETWEEN CHANGES OF BRAIN ACTIVATION IN ΔEXCLUSION—MICRO-REJECTION > OVERINCLUSION—INCLUSION AND PERCEIVED PERCENTAGE OF THE THROWS**					
L. visual cortex (19)	56	−6	−26	26	4.64

Notes: L, left; t: t-value. Brodmann's area is provided in parentheses.

**Table 6:** **Regression analyses between brain activation and surprise**.

**Brain region**	***x***	***y***	***z***	**size**	***t***
**POSITIVE CORRELATION BETWEEN CHANGES OF BRAIN ACTIVATION IN ΔEXCLUSION—MICRO-REJECTION > OVERINCLUSION—INCLUSION AND SURPRISE IN ΔEXCLUSION—OVERINCLUSION**					
R. visual cortex (19)	36	−62	4	38	5.46
R. hippocampus	44	−38	−10	17	4.67
R. caudate nucleus	16	−10	40	31	4.49
**NEGATIVE CORRELATION BETWEEN CHANGES OF BRAIN ACTIVATION IN ΔEXCLUSION—MICRO-REJECTION > OVERINCLUSION—INCLUSION AND SURPRISE IN ΔEXCLUSION—OVERINCLUSION**					
L. SPL (7)	−42	−18	50	17	5.35
	−40	−42	40	13	4.14
L. LPFC (44)	−34	34	18	22	4.72
R. PMC (6)	32	−2	38	18	5.07

Notes: L, left; R, right; SPL, superior parietal lobule; LPFC, lateral prefrontal cortex; PMC, primary motor cortex; t: t-value. Brodmann's area is provided in parentheses.

## Discussion

The main goal of our study was to identify the brain regions that are sensitive to social exclusion, by examining the effects of both exclusion and overinclusion. We used an event-related continuous block design to operationalize these social scenarios. Two sets of findings emerged as important and informative for our understanding of social exclusion experiences: (1) both the dACC and rVLPFC were activated during exclusion events after controlling for expectancy violation (i.e., exclusion-related event > inclusion-related event); and (2) increasing rVLPFC activity was associated with decreasing self-rated social pain, whereas dACC activity was not associated with self-rated social pain.

### Subjective effects of social exclusion and overinclusion

Participants in the present study felt more social pain during exclusion, as was the case with past studies using the same design (Bolling et al., 2011b) or the original Cyberball design featuring longer inclusion and exclusion trial blocks (e.g., Williams et al., 2000; Onoda et al., 2009; Yanagisawa et al., 2011a,b). Social exclusion is so highly baneful for primates that members of these species are quite sensitive to its potential effects (Kling et al., 1970; Silk et al., 2003). In human society, exclusion can cause various difficulties such as loss of contact with important others or groups (Williams et al., 2000; Eisenberger and Lieberman, 2004; Macdonald and Leary, 2005). Our findings strongly suggest that people can detect and experience aversive feelings even at the slightest hint of social exclusion.

We did not find a social pain difference between inclusion and overinclusion conditions. This finding is consistent with the notion that being overincluded is not a more positive experience than being included to a more appropriate or typical degree (Williams et al., 2000). Previous findings indicate that a negative event is subjectively more potent and of higher salience than its positive equivalent, when opposing negative and positive events are of an equal objective magnitude (e.g., Taylor, 1991; Rozin and Royzman, 2001). That is, people react more strongly to the negative event when they encounter positive or negative events of similar magnitude. Diverse negative interpersonal phenomena are encountered in everyday life, including rejection, discrimination, ostracism, betrayal, and stigmatization (Smart Richman and Leary, 2009), whereas overinclusive situations appear to be relatively rare and unnatural. In line with this notion, we found that participants felt slightly more surprise during overinclusion than during exclusion. Our findings imply that being overincluded is not a more positive experience than being appropriately included, but such an experience does make participants feel conspicuous. Moreover, participants reported that more throws went to them during overinclusion as compared to exclusion. Note that both conditions significantly differed from the midpoint (i.e., 50%), and the absolute difference values between the scores for both conditions did not differ from the midpoint. These findings indicate that both exclusion and overinclusion make participants feel conspicuous, as has been found in previous studies (Williams et al., 2000; van Beest et al., 2011).

### Neural effects of social exclusion and overinclusion

The most important finding of the present study was that dACC activity in response to exclusion events appears to reflect the detection of social exclusion rather than expectancy violation alone. This finding is partly consistent with past findings, such that the dACC is activated in response to social exclusion (Eisenberger et al., 2003). dACC activity in response to social exclusion has previously been conceptualized as a neural “alarm system” (Eisenberger and Lieberman, 2004). Eisenberger and Lieberman (2004) argued that two systems are needed for adequate operation of the alarm system. The first is a discrepancy monitoring system, which serves to detect deviations from desired standards. The second is a sounding mechanism that signals a problem that needs to be addressed. The dACC's discrepancy-detection function is considered to be associated with the detection of social exclusion, whereas social pain is thought to be the product of the sounding system. Our findings seem to show that dACC activation reflects the former component. Note that overinclusion did not activate the dACC, indicating that the dACC activation found in previous social exclusion studies is not due solely to expectancy violation. Our findings suggest that dACC activity plays an important role in the detection of exclusion.

In contrast to prior work, we did not observe vACC activity in response to exclusion (Bolling et al., 2011b). This region is also responsive to one's perceptions of emotional support (Coan et al., 2006; Onoda et al., 2009). Furthermore, clinical research has identified greater levels of vACC activity in depression (e.g., Chen et al., 2007; Yoshimura et al., 2010). The vACC may therefore play a crucial role in the experience of social exclusion. However, some previous studies have also shown that the vACC is involved in more positive affective processes, such as social acceptance (Somerville et al., 2006), lower sensitivities to facial rejection (Burklund et al., 2007), and optimism (Sharot et al., 2007). The vACC seems to be involved in emotional processing regardless of the specific valence of the experienced emotion. We could not directly observe vACC activity, because this study was designed to compare overinclusion-related events, which have a relatively positive emotional valance, and exclusion-related events, which have a relatively negative emotional valance.

It must also be noted that the rVLPFC was activated in response to exclusion-related events, such that activity in this region was negatively correlated with social pain. Activation in this region is associated with the regulation or inhabitation of negative affect (Hariri et al., 2000; Small et al., 2001; Petrovic et al., 2002) as well as pain-induced distress (Eisenberger et al., 2003, 2007; Yanagisawa et al., 2011a,b). The rVLPFC seems to be involved in the regulation of social pain, and our finding of a relationship between event-related rVLPFC activity and overall subjective social pain appears to be novel. Our findings imply that neural activity in response to exclusion may modulate feelings of social pain.

With regard to dACC and rVLPFC activation in response to exclusion-related events, overinclusion-related events did not give rise to activation in the neural regions previously associated with receiving positive social feedback, such as the ventral striatum (VS) (e.g., Izuma et al., 2008). There are several possible reasons for this. First, overinclusion may not be a positive event. Our subjective rating findings indicate that overinclusion events are not experienced as more positive than inclusion events, but do make participants feel conspicuous, as found in previous studies (Williams et al., 2000). This may have rendered it impossible to observe specific reward-related neural activities in response to overinclusion. A second possibility is that exclusion events may reduce reward processing. Research showing VS activity in response to positive social feedback has included only positive and neutral feedback trials, with no negative feedback trials being used (Izuma et al., 2008). The fact that we also used negative events (i.e., exclusion) may have reduced the impact of rewarding experiences associated with positive social feedback.

### Limitations and future directions

Several limitations of this study should be noted. First, we used a short continuous block design and subdivided events to permit analysis of different trial blocks. Because of this, it is possible that our design was not sufficient to evoke robust feelings of exclusion. However, the fact that we observed exclusion-related neural activity and increases in subjective social pain suggests that our design was adequate to produce the phenomena of interest. Second, we examined the relationship between event-related neural activities and overall subjective feelings. It is possible that the event-related design might be less optimal for studying relationships that involve self-report ratings, because these ratings might capture affective responses associated with the overall exclusion experience instead of single trials. Our study design made it difficult to assess online subjective distress during exclusion, given that assessment process would make the task unnatural and perhaps change its meaning. Future research could assess online distress using psychophysiological approaches such as facial electroencephalogram. Third, we were unable to test for gender effects, as there were only three males in our study. While we did not expect any significant gender effects, as previous social exclusion studies have not revealed much in the way of such effects, we cannot eliminate the possibility that such effects occurred in our sample. Finally, it has been suggested that adolescent changes in social orientation coincide with structural and functional changes in the brain (Nelson et al., 2005; Blakemore, 2008). In exclusion studies, for example, rVLPFC activation was higher in adults as compared to adolescents during social exclusion (Bolling et al., 2011a; Sebastian et al., 2011). On the other hand, the vACC seems to play an important role in emotional processing of social exclusion among adolescents (Masten et al., 2009, 2011). Future research could examine neural responses in adults and adolescents in order to track how the neural alarm system developmentally changes.

## Conclusion

The present study revealed that dACC and rVLPFC activity might represent a neurocognitive index of social exclusion processing. The dACC could be involved in the detection of social exclusion, whereas the rVLPFC plays an important role in the regulation of social pain. This dual mechanism can be considered to be one possible foundation of the neurobiology of social exclusion.

### Conflict of interest statement

The authors declare that the research was conducted in the absence of any commercial or financial relationships that could be construed as a potential conflict of interest.
